# Eye movement defects in KO zebrafish reveals *SRPK3* as a causative gene for an X-linked intellectual disability

**DOI:** 10.21203/rs.3.rs-2683050/v1

**Published:** 2023-03-20

**Authors:** Yu-Ri Lee, Mervyn G. Thomas, Arkaprava Roychaudhury, Cindy Skinner, Gail Maconachie, Moira Crosier, Holli Horak, Cris S. Constantinescu, Tae-Ik Choi, Jae-Jun Kyung, Tao Wang, Bonsu Ku, Bernard N Chodirker, Michael F. Hammer, Irene Gottlob, William H. J. Norton, Albert E. Chudley, Charles E. Schwartz, Cheol-Hee Kim

**Affiliations:** 1 Department of Biology, Chungnam National University, Daejeon 34134, South Korea; 2 KM Convergence Research Division, Korea Institute of Oriental Medicine, Daejeon 34054, South Korea; 3 The University of Leicester Ulverscroft Eye Unit, Department of Neuroscience, Psychology and Behaviour, University of Leicester, Leicester, UK; 4 Greenwood Genetic Center, Greenwood, SC 29646, USA; 5 Division of Ophthalmology and Orthoptics, Health Science School, University of Sheffield, UK; 6 Human Developmental Biology Resource, Biosciences Institute, Faculty of Medical Sciences, Newcastle University, Newcastle upon Tyne NE1 4EP, UK; 7 Department of Neurology, University of Arizona, Tucson, AZ 85724, USA; 8 Academic Unit of Mental Health and Clinical Neuroscience, University of Nottingham, NG7 2UH, UK; 9 Cooper Neurological Institute and Cooper Medical School of Rowan University, Camden, NJ 08013, USA; 10 McKusick-Nathans Department of Genetic Medicine, Johns Hopkins University, Baltimore, MD 21287, USA; 11 Disease Target Structure Research Center, Korea Research Institute of Bioscience and Biotechnology, Daejeon 34141, South Korea; 12 Department of Pediatrics and Child Health, Max Rady College of Medicine, Rady Faculty of Health Sciences, University of Manitoba, Winnipeg, Manitoba R3A 1R9, Canada; 13 BIO5 Institute, University of Arizona, Tucson, AZ 85721, USA; 14 Department of Genetics and Genome Biology, University of Leicester, Leicester LE1 7RH, UK; 15 Department of Pediatrics and Human Development, College of Human Medicine, Michigan State University, Grand Rapids, MI 49503, USA; 16 These authors contributed equally: Yu-Ri Lee, Mervyn G. Thomas, Arkaprava Roychaudhury.

## Abstract

Intellectual disability (ID) is a common neurodevelopmental disorder characterized by significantly impaired intellectual and adaptive functioning. X-linked ID (XLID) disorders, caused by defects in genes on the X chromosome, affect 1.7 out of 1,000 males. Employing exome sequencing, we identified three missense mutations (c.475C>G; p.H159D, c.1373C>A; p.T458N, and c.1585G>A; p.E529K) in the *SRPK3* gene in seven XLID patients from three independent families. Clinical features common to the patients are intellectual disability, agenesis of the corpus callosum, abnormal smooth pursuit eye movement, and ataxia. SRPK proteins are known to be involved in mRNA processing and, recently, synaptic vesicle and neurotransmitter release. In order to validate *SRPK3* as a novel XLID gene, we established a knockout (KO) model of the *SRPK3* orthologue in zebrafish. In day 5 of larval stage, KO zebrafish showed significant defects in spontaneous eye movement and swim bladder inflation. In adult KO zebrafish, we found agenesis of cerebellar structures and impairments in social interaction. These results suggest an important role of *SRPK3* in eye movements, which might reflect learning problems, intellectual disability, and other psychiatric disorders.

## INTRODUCTION

Intellectual disability (ID) affects approximately 1–3% of the general population [1]. Males exceed females in the ID population by 20–30%, likely due to an enrichment of genes on the X-chromosome that are required for neurodevelopment. X-linked ID (XLID) disorders, resulting from hemizygous variants, contribute significantly to the male ID population [2]. These conditions are chronic and present from birth, lasting throughout the lifetime of the affected individuals. Consequently, this group of disorders requires long-term family involvement, medical care, and social services, which impose a significant burden on both the families and society. Efforts by research groups worldwide have identified 145 XLID genes contributing to 114 XLID syndromes and 63 non-syndromic XLID entities. Exome sequencing has accelerated mutational analysis of the coding regions of the X-chromosome and identified 28 of the 145 XLID genes in the past decade [3]. However, ID is characterized by significant genotypic and phenotypic heterogeneity. Recent estimates suggest that the number of genes linked to ID has risen to over 1,500 genes [4]. The availability of a complete sequence for the X chromosome, coupled with the capability of next-generation sequencing and functional analysis, presents an exciting opportunity to identify the genetic basis for at least one-third of this group of disorders for which the causative gene has not yet been identified.

Through exome sequencing, we initially identified a missense mutation in the *SRPK3* gene (serine/arginine-rich protein-specific kinase 3) in an XLID family. The mutation was maternally inherited from a Manitoba mother of Anglo-Saxon heritage. Subsequently, we became aware of a second mutation identified by the Undiagnosed Disease Program (UDP) at NIH, USA. Given the presence of two missense mutations in *SRPK3*, we conducted a search of a database generated by our previous Sanger X-chromosome resequencing project [5], which revealed a third missense mutation. The combination of these three missense mutations made *SRPK3* a compelling candidate gene for in-depth analysis as a novel XLID gene. SRPK3 is known to specifically phosphorylate serine-arginine (SR) proteins which act as splicing factors [6]. Phosphorylation is required for SR proteins to enter the nucleus and play a role in alternative splicing of pre-mRNA, mRNA export, and other processing events. Recently, new functions of SRPK2 were reported in synaptic vesicle and neurotransmitter release [7]. Although our bioinformatic analysis predicted that the three variants were likely pathogenic and related to XLID phenotypes in the families, further investigation was required to confirm these predictions and elucidate the molecular mechanisms underlying the observed XLID phenotype.

In order to investigate SRPK3 as a novel XLID gene, a knockout zebrafish model of *srpk3* was generated and evaluated at different developmental stages to understand how SRPK3 deficiency adversely affected its function *in vivo*.

## MATERIAL AND METHODS

Further details about the procedures are described in the Supplementary Methods.

The human subject research protocol for the study was approved by the Institutional Review Boards (IRBs). Informed consent was obtained from each study patient and/or their parents or legal guardians. Patients enrolled in the Greenwood Genetic Center XLID study were evaluated by clinical geneticists and other specialists and underwent comprehensive laboratory studies for ID. All patients were found to have a normal karyotype and negative molecular testing for Fragile X syndrome. Given that each center independently identified *SRPK3* as the novel candidate gene for ID, the enrichment kits used and sequencing protocols varied between centers.

### Family 1 (K8765, GGC)

Patients with XLID and normal control males were recruited at Greenwood Genetic Center (GGC), USA. X-chromosome exome sequencing on probands from XLID families was conducted using a TruSeq Genomic DNA Library Preparation kit, a SureSelect Target Enrichment kit, and the HiSeq2000. Bowtie2 and Unified Genotyper were used for alignment, base recalibration, and variant calling. Sanger sequencing was used for validation, segregation analysis, and polymorphism studies.

### Family 2 (UDP, NIH)

After identification of the first mutation, subsequently, we became aware of a second mutation identified by the Undiagnosed Disease Program (UDP) at NIH, USA. Drs. Camilio Toro and Neal Boerkoel (NHGRI, USA) had kindly provided information on the second mutation, which allowed us to compare the phenotypes.

### Family 3 (UAGC)

Clinical information on males of family 3 had been provided by the University of Arizona Genomics Core (UAGC), USA. Whole exome sequencing was performed twice on four samples from the family using different enrichment kits. Variants were annotated and candidate X-linked variants were identified by comparing the genotype of the male proband to that of the mother and father. A prioritizing system was implemented to identify true X-linked variants, and those within the highest priority level were validated by Sanger sequencing. Variants with a frequency <3% in the ESP were also screened for. *De novo* mutations in the proband that were not observed in the 1000 Genomes or ESP datasets were also examined. SRPK3 mutations are reported as per HGVS guidelines against the accession ID: ENST00000370101.8

### Expression studies for protein and mRNA

Human embryonic samples were collected with appropriate maternal consents and ethical approval by NRES Committee North East-Newcastle & North Tyneside 1. Spatiotemporal SRPK3 brain expression was investigated by immunohistochemistry on human embryonic tissue as described previously [8]. The embryonic development stage was determined by external morphology assessment as previously described [9,10]. We analyzed the developmental transcriptome datasets from the BrainSpan project [11,12], specifically examining spatiotemporal expression of *SRPK3* mRNA in the developing prenatal and post-natal brain. Details regarding transcriptome profiling is available at www.brainspan.org.

### Zebrafish husbandry

A line of wild-type zebrafish (*Danio rerio*) maintained in our animal facility were reared under specific environmental conditions. All experiments were conducted with approval from the Institutional Animal Care and Use Committees (IACUC) of Chungnam National University (202012A-CNU-170). Zebrafish used in the experiment were obtained from the Zebrafish Center for Disease Modeling (ZCDM; South Korea).

### Whole mount *in situ* hybridization and histological analysis

Whole-mount *in situ* hybridization was performed using a protocol described previously [13]. The fixed embryos were dehydrated with MeOH, rehydrated, and permeabilized with Proteinase K. RNA probe was synthesized *in vitro* using a cloned amplicon and then dissolved in HYB+ solution for use in the experiment. The antisense RNA probe was added to the embryos and allowed to hybridize overnight. The larvae were then washed and incubated with an anti-DIG Fab fragment conjugated with alkaline phosphatase. For histological analysis, paraffin sections of the adult zebrafish brain were stained with H & E staining.

### Generation of *srpk3* KO zebrafish model using CRISPR/Cas9

The gene sequences for *srpk3* were obtained from the NCBI database, and primers were designed for *in vitro* transcription of sgRNAs targeting *srpk3*. Cas9 expression vector pT3TS-nCas9n was used to synthesize Cas9 mRNA, which was injected along with sgRNAs into one-cell stage zebrafish embryos. Mutations were validated using T7E1 assay, and founder (F0) fish were raised to adulthood and out-crossed with wild-type zebrafish to generate germ line mutations. The F1 generation was raised to adulthood, and individuals with the heterozygous genotype were in-crossed to produce stable *srpk3* KO zebrafish lines (F2) [14].

### Analysis of spontaneous eye movements in KO zebrafish

Zebrafish larvae were maintained at a constant temperature of 28.5°C until they reached 5 dpf, which is a stage commonly used for spontaneous eye movement analysis in zebrafish. Zebrafish larvae at 5 dpf were mounted on a petri dish using 2% low melting agarose and a 3-minute video of their eye movements was recorded using a brightfield microscope. The recorded video was then analyzed using MATLAB software to track and analyze the eye movements of the zebrafish larvae [15].

### Social interaction assay in adult KO zebrafish

In the social interaction assay [16], behavior of zebrafish was observed in a tank divided into two sections. One section was designated for the cue fish and the other for the tester fish. The tank was divided into four equal chambers (zone 1, 2, 3, and 4); the zone nearest to the social cue was designated zone “1”, the second nearest zone “2”, the third zone “3”, and the last zone “4”. During the experiment, three adult zebrafish were added as cue fish and a single *srpk3* KO sibling was added as the tester fish. A video was taken for 15 minutes and the behavior was analyzed using Ethovision XT software.

### Statistical analysis

Statistical analysis was performed using Graphpad Prism 8. Data were expressed as mean ± standard error of the mean (SEM) with a p value of < 0.05 being considered significant. For parametric measures of two groups, a two-tailed T-test was performed and for more than two groups, One-Way ANOVA with Tukey’s post-test was performed.

## RESULTS

### Patient information and bioinformatic analysis of SRPK3 mutations

Clinical information on males with the p.H159D and p.E529K mutations had been provided by the Department of Pediatrics and Child Health (University of Manitoba, Canada) and the University of Arizona Genomics Core (UAGC, USA) respectively as part of our large XLID study. Learning difficulties and ID were noted in all patients. Two families, H159D and T458N, were noted to have brain abnormalities: agenesis of the corpus callosum and cerebellar atrophy, respectively. These same two families had abnormal speech and all three families exhibited mild ID. Thus, similarity of the phenotypes was consistent with the mutations in *SRPK3* being relevant to XLID in the three families. As ID-related features, learning difficulties and/or delayed language skills, were shown in all affected patients, eye movement-related phenotypes, such as abnormal smooth pursuit, convergence insufficiency, lazy eye, and/or poor attention span, were also clinically described in the four patients (Table S1). Bioinformatic analysis predicted that the p.H159D, p.T458N, and p.E529K mutations were very likely disease causing. All three missense mutations segregated in their respective families. Among these variants, c.475C>G; p.H159D was identified in Family 1 by X-chromosome exome sequencing (PMID25679214); c.1373C>A; p.T458N was identified in Family 2 at NHGRI; c.1585G>A; p.E529K was identified in Family 3. The minor allele frequency (MAF) of the reported *SRPK3* variants was evaluated using gnomAD. Our analysis revealed that the c.1585G>A variant exhibited a very low MAF (MAF=0.0001), whereas c.475C>G and c.1373C>A variants were not detected in the gnomAD dataset. All three variants had high CADD scores of: 24.8 (c.475C>G), 24.4 (c.1373C>A), and 25.5 (c.1585G>A); a high CADD score indicates a greater likelihood that these variants are deleterious.

### Family 1 and 2: p.H159D, p.T458N

**Fig 1:**
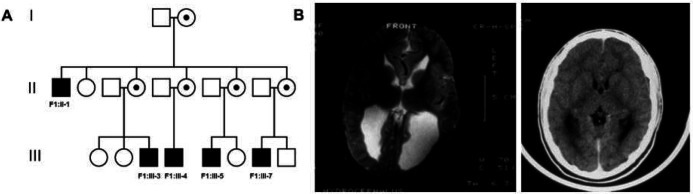
Pedigree of Family 1 demonstrating X-linked pattern of inheritance. **A** The first mutation in the *SRPK3* gene (c.475C>G; p.H159D) was identified in five affected males (F1:II-1, F1:III-3, F1:III-4, F1:III-5, F1:III-7) from a well-established XLID family. **B** Brain imaging were performed on four of the five affected males. A representative MRI image of the patient F1:III-4, demonstrating a dilatation of the occipital horns.

The first mutation c.475C>G; p.H159D in Family 1 was maternally inherited from a Manitoba mother of Anglo-Saxon heritage (Fig. 1A). Each male presented at an early age with delayed motor, social, adaptive and language skills. Family 1 was referred to the genetics clinic in Winnipeg, Manitoba due to cognitive impairments in five males, ranging from mild to moderate severity. The pattern of inheritance was consistent with an XLID; however, the etiology remained unknown. Brain imaging (X-ray, CT, and/or MRI scans) were performed on four of the five affected males, revealing irregular and asymmetrically dilated lateral ventricles in all four males (Fig. 1B). Three males demonstrated dilatation of the occipital horns (colpocephaly). Additionally, evidence of various degrees of agenesis and/or dysgeneis of the corpus callosum were observed in all four males. In one male, there was moderate to severe white matter bulk loss; for example, patient F1:II-1 had a large head with possibility of an arrested hydrocephalus whereas patient F1:III-5 had mild delay in gross motor milestones – sitting at 8 months, walking at 18 months and not speaking until after 3 years – and having a poor attention span. Patient F1:III-4 also had a very short attention span. His developmental profile was scattered and there was a delay in the cognitive area. However, none of the four affected males exhibited facial dysmorphic features or other visible malformations; three of the males had macrocephaly. We excluded commonly known forms of X-linked disorder with these brain image findings during their assessments conducted between 1984 and 2013, when this study was initiated. The second mutation (p.T458N) in Family 2 was identified by the Undiagnosed Disease Program (UDP) at NHGRI, NIH.

### Family 3: p.E529K

The proband (F3:III-1) in Family 3 presented with ID, muscle hypotonia, and ataxia. His maternal uncle, hemizygous for the *SRPK3* variant, also had similar phenotypical characteristics. The proband’s mother was heterozygous for the *SRPK3* variant and asymptomatic (Fig. S1). Brain MRI was performed only on the proband, revealing a bilateral and symmetric prominence of the convexity sulci posteriorly with a mild posterior-to-anterior gradient. Mild bilateral symmetric reduction in parietal gyral white matter volumes was observed. Furthermore, a loss of volume within the posterior body of the corpus callosum was identified likely attributed to a reduction in volume of traversing fibers in this region.

### CNS expression of SRPK3 in human and zebrafish

**Fig 2:**
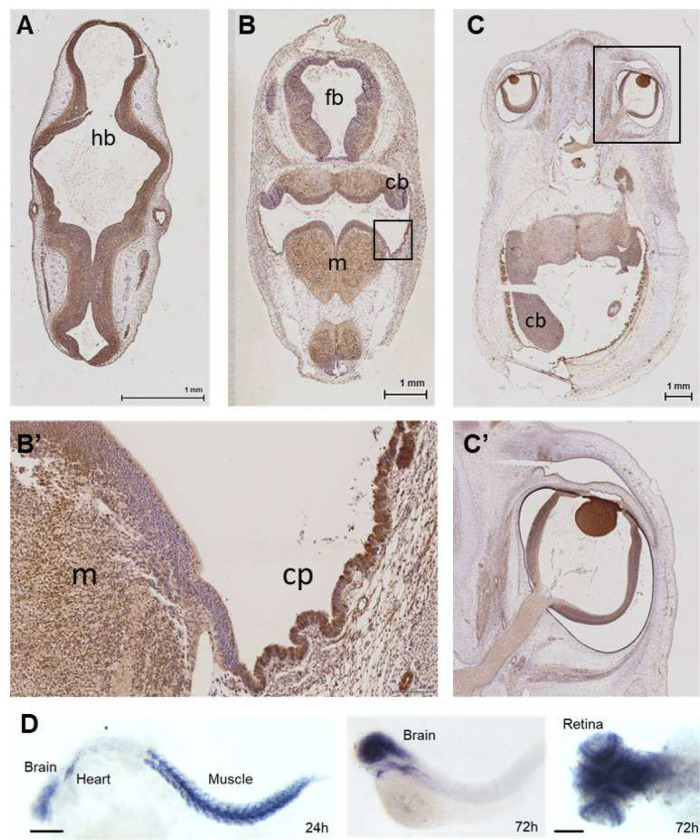
CNS expression of SRPK3 in human and zebrafish embryos. **A** At Carnegie Stage 15 (CS15), immunohistochemistry results showing widespread staining of SRPK3 within the hindbra in (hb). **B** At CS 19 post mitotic expressionis noted in the forebrain (fb), cerebellum (cb) and med ulla (m). **C** At CS23 expression is noted in cerebellum. **B’, C’** High magnification images showin g intense staining of choroid plexus (cp) epithelium and expression within the cerebellum (cb), m edulla (m), and eye. **D** Spatiotemporal expression of *srpk3* in developing zebrafish embryo show ing transcripts of *srpk3* being present at 24 hpf in the brain, heart and muscles, and in the brain and retinal layers at 72 hpf. Scale bars: 1 mm for A-C, 100 μm for D.

Experiments confirmed the expression of SRPK3 in the human fetal brain and heart. At Carnegie stage (CS) 15 [9] we identified widespread expression of SRPK3 within the developing brain, retina, spinal cord, heart muscle, and limbs. The expression is mostly post mitotic and not nuclear. At stage CS19 expression was observed in the cerebellum and medulla with strong expression in the hindbrain choroid epithelium. Interestingly, at CS23 a strong expression was identified in the choroid epithelium. Comparatively weaker expression was seen in the cerebellum, pons and medulla. At both stages, expression was mostly post mitotic but both nuclear and cytoplasmic staining was seen (Fig. 2A–C’). The RNA-Seq dataset revealed that SRPK3 mRNA expression increased with age with the highest level of expression being noted within the cerebellum (Fig. S2).

To examine the spatiotemporal expression profiles of *srpk3* during zebrafish development, whole-mount *in situ* hybridization was performed. Robust expression of *srpk3* transcripts was detected at an early stage of approximately 24 hpf, with specific expression observed in the brain, heart, and muscle. At 72 hpf, the expression remained in the brain region and central neural tissues. Very specific expression could be observed in the retinal layers of zebrafish larvae.

Interestingly, SRPK3 was expressed at a high level in developing retina both in human and zebrafish embryos (Fig. 2C–D), implying a conserved role of SRPK3 function in the eye.

### Molecular modeling of the three mutations implicated in XLID

**Fig 3:**
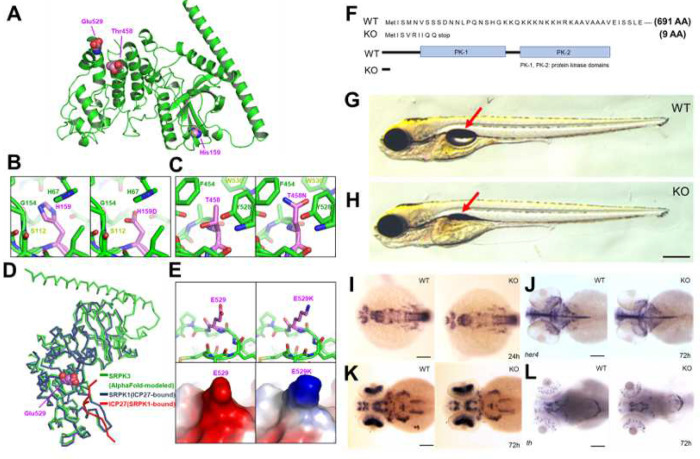
Molecular modeling of SRPK3 and generation of *srpk3* zebrafish KO line. **A-E** Molecular modeling of SRPK3 variants. **A** Side chains of three amino acids involved in XLID are indicated as spheres on the SRPK3 structure modeled using AlphaFold. Unstructured loop regions of SRKP3 are omitted for clarity, including residues 1–46 and 310–385. **B, C** Interior misfolding due to H159D and T458N variations. SRPK3 wild-type (left) and the H159D (**B**, right) or T458N (**C**, right) mutant form modeled based the AlphaFold structure prediction are shown together. **D** Structural superposition of the AlphaFold-modeled SRPK3 (green) onto the crystal structure of ICP27 (red)-bound SRPK1 (Navy; PDB code 6FAD). Side chain atoms of SRPK3 Glu529 are presented as spheres. **E** Alteration of protein shape and surface charge due to E529K variation. SRPK3 wild-type (left) and the E529K mutant form (right) are shown as sticks and loops (top) or electrostatic surface representation (bottom). **F-L** Characterization of *srpk3* zebrafish KO line. **F** Disruption of protein domains in KO zebrafish while wild type zebrafish show intact *srpk3* protein. **G, H** KO zebrafish fail to develop swim bladder inflation at 5 dpf when compared to wild type siblings (red arrow). **I-L** Expression analysis of neuronal markers (*neurogenin 1, her 4*, *islet 1* and *th*) in developing zebrafish embryos. Scale bars are 100 μm.

Effects of the three XLID-associated missense mutations (H159D, T458N, and E529K) on SRPK3 structure was modeled by Alphafold (https://alphafold.ebi.ac.uk/entry/Q9UPE1) (Fig. 3A–E). H159D and T458N substitutions could cause protein misfolding because of their location on the interior of SRPK3 and their side chains playing a structural role in the maintenance of protein folding (Fig. 3B and 3C). For instance, while the imidazole ring of His159 mediates 72 atom–atom contacts within the distance of 4.5 Å, the corresponding contacts mediated by the side chain carboxyl group decreased to 39 contacts due to H159D substitution (Fig. 3B). The T458N mutation appeared to cause steric hindrance with neighboring bulky residues such as Phe454, Tyr528, and Trp530, presumably resulting in disturbance of SRPK3 protein folding (Fig. 3C). In contrast, the side chain of Glu529 was exposed to the outside of the protein, and thus its substitution to lysine seems not to have affected protein stability. Instead, we noticed that SRPK1 and SRPK3 share a high structural similarity with each other with a root-mean-square deviation value of 0.73 Å over 343 aligned Cα atoms, and Glu529 was found to be located adjacent to SRPK1-bound protein fragment in the superposed model (Fig. 3D). Therefore, we hypothesize that E529K substitution led to alteration of protein shape and surface charge which ultimately affected protein–protein interaction-mediated target protein recognition of SRPK3 (Fig. 3E).

### Generation of *srpk3* KO zebrafish lines using CRISPR-Cas9 system

The human homologue of *SRPK3* is located on Xq28, while in zebrafish, *srpk3* homolog is located on chromosome 8. We performed a ClustalX 2.1 alignment of the amino acid sequences of both homologues, which revealed a high degree of conservation (Fig. S3). Specifically, the human homologue contains 567 amino acids, while the zebrafish homologue contains 701 amino acids. Missense mutations H159D, E529K, and T458N are marked with red arrows in Fig. S3A. To explore the functional consequences of *srpk3* mutations in zebrafish, CRISPR-Cas9 technology was utilized to generate KO zebrafish lines. Target sites at the exon of s*rpk3* were designed and the induced mutations were analyzed by sequencing. The different KO mutant forms obtained were +27bp, +1bp, −3bp, −8bp, −4bp, and −7bp (Fig. S3B). We used a s*rpk3* KO zebrafish line which has an in/del mutation predicted to produce truncated proteins (Fig. 3F)

### Morphological analysis of *srpk3* KO zebrafish at early developmental stages

*srpk3* KO zebrafish showed normal development at early embryonic stages. Evaluation of cell death was determined via vital dye acridine orange staining that is often used as a marker of apoptotic cells in zebrafish (Fig. S5). Next, morphological defects in larval zebrafish at 5 dpf, including body length, pigment patterns, and eye size were analyzed. Although we did not find any significant defects in these aspects, we observed that the majority of KO zebrafish failed in swim bladder inflation (Fig. 3G, H). Swim bladder inflation is an early marker for survival in larval zebrafish, as it is a vital organ which enables fish to swim. In total, 2,367 larvae from 10 independent clutches were examined. The incidence of swim bladder defects was decreased from 23.4% to 4.3% in subsequent generations (Table S2). 6 out of 73 (8%) adult *srpk3* KO zebrafish survived into adulthood (Fig. S4). Therefore, the lack of swim bladder inflation in *srpk3* KO zebrafish was considered a significant feature which prompted molecular marker analysis.

### Expression of neuronal markers in *srpk3* KO zebrafish

By whole-mount *in situ* hybridization, mRNA expression of early neuronal markers, such as *neurogenin1* and *her4*, was examined in wild type and KO *srpk3* zebrafish siblings. No significant change was observed in the expression level of these molecular markers in the homozygous KO zebrafish compared to the wild type. We also analyzed the motoneuron marker *islet 1* and the dopaminergic neuronal marker *tyrosine hydroxylase* (*th*) in KO zebrafish, but again we did not observe significant changes in homozygous KO zebrafish compared to its wild type siblings (Fig. 3I–L). Additionally, we examined the expression of other neuronal markers (*phox2b, ascl1, dlx2*), cell cycle marker (*ccdn1*), and muscle markers (*bin1b, ttnb*) but did not detect significant differences in expression levels at early developmental stages between KO and wild type siblings (Fig. S6). Thus, the introduction of genome-scale analysis is needed to identify underlying molecular mechanisms in further studies.

### *srpk3* KO zebrafish show defects in spontaneous eye movements

**Fig 4:**
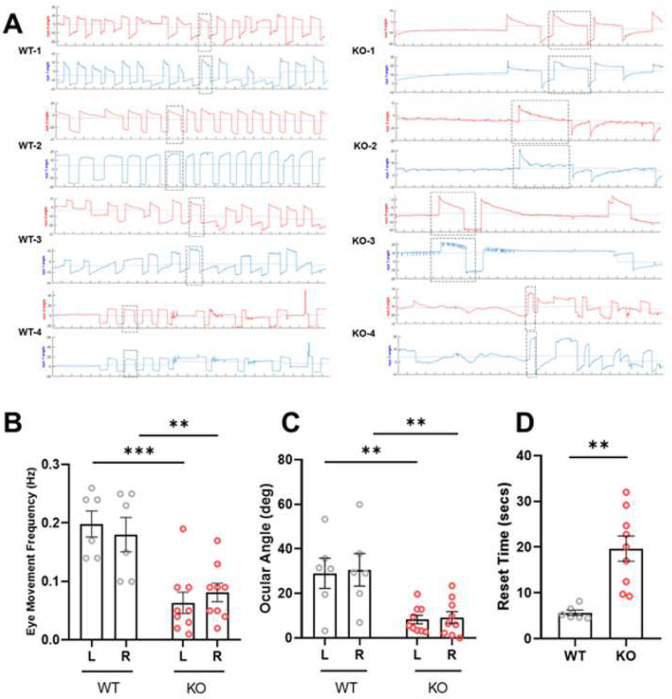
Analysis of spontaneous eye movements in KO zebrafish. **A** Individual representation of spontaneous eye movement in wild type (WT) and *srpk3* KO siblings. Red lines represent right eye and blue lines represent left eye of 5 dpf zebrafish larvae. **B** Eye movement frequency in the left (L) and right (R) eye of *srpk3* KO zebrafish when compared to its WT siblings. **C** Ocular angle significantly decreases in both left and right eye in KO zebrafish, compared to WT siblings. **D** *srpk3* KO zebrafish take significant longer time to reset in slow phase when compared to their WT siblings during spontaneous eye movement. Video recording of WT (n = 6) and KO (n = 9) zebrafish for 3 minutes. All data represented as mean ± SEM. *p < 0.05, ** p < 0.01, *** p < 0.001 by t test.

Spontaneous eye movements in all vertebrates are primarily characterized by saccades in one direction followed by a fixation period and a saccade in the opposite direction [17]. To investigate the role of *srpk3* in neurodevelopmental disorders, we analyzed spontaneous eye movements in 5 dpf zebrafish larvae. In wild type *srpk3* siblings, yoked eye movements occurred such that both eyes moved in a synchronous unidirectional pattern [18], which is important for maintaining spatial connection in the visual spectrum [19]. However, *srpk3* KO siblings exhibited significant reduction in eye movement frequency (Fig. 4; Movie S1), indicating its involvement in maintaining the pattern of movement. Although most wild types displayed robust synchronized movements in both eyes, KO zebrafish showed a reduction in ocular angle (Fig. 4C). To further investigate this, we analyzed one spontaneous movement pattern in WT and KO zebrafish and found a significant reduction in reset time (Fig. 4D). Next, we examined whether KO zebrafish lost their visual acuity. In a color preference test, which we had previously developed [20], KO zebrafish showed normal visual activity of color preference at 5 dpf, compared to WT siblings (Fig. S7). Taken together, we concluded that *srpk3* KO zebrafish have significant disruptions in spontaneous eye movement, which may contribute to intellectual and learning disabilities [21–23].

Recently, eye movement defects reported in other neurological disease models in zebrafish – such as down syndrome cell adhesion molecule-like 1 (*dscaml1*), which is thought to be involved in autism spectrum disorder (ASD) and cortical abnormalities – exhibited behavior analogous to congenital ocular motor apraxia [24]. Similarly, *taf1* [25] and *katnal2* [26], which are associated with intellectual disability, exhibited eye defects in their KO zebrafish. Zebrafish *belladonna/(lhx2)* mutants showed involvement in congenital nystagmus [27,28] and agenesis of the corpus callosum in *Lhx2* mutant mice [29].

### *srpk3* KO zebrafish show impaired social interaction

**Fig 5:**
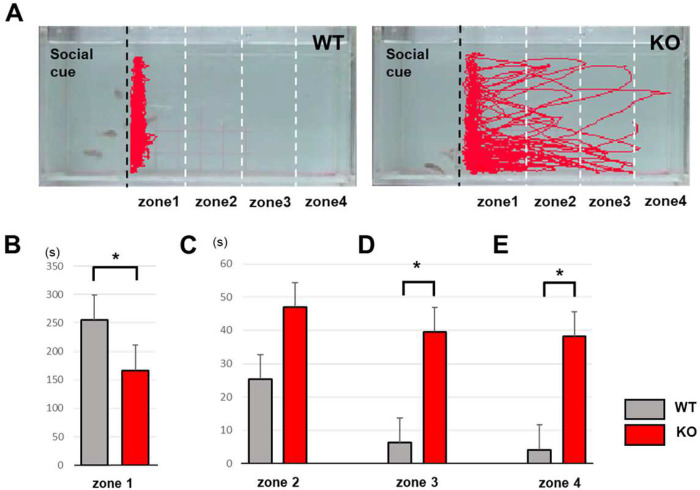
*srpk3* KO adult zebrafish show impairment in social interaction. **A** Video tracking of 5 minutes movements (10–15 minutes) in wild type adult and KO zebrafish, showing difference in social interaction with social cue fish. Three wild type fish were used as social cue and one as a tester fish. Black dashed line indicates the position of the transparent separator in the water tank. White dashed lines show the boundaries of four different zones. **B** Duration time for the tester fish in each zone. All data represented as mean ± SEM. *p < 0.05 by t test.

One of the main indicators for intellectual disability is social interaction, for which we had previously established a behavioral assay in zebrafish [16]. Since social interaction-related features were also observed in the four affected patients (Table S1) with poor attention span in two patients (F1:III-4 and F1:III-5) and eye movement defects in two patients (F2 and F3:III-1), we challenged *srpk3* KO zebrafish to social interaction testing (Fig. 5). As a result, we observed that *srpk3* KO adult zebrafish displayed defects in social interaction, especially at the 10–15-minute time point (Fig. 5A). We analyzed the behavior of fish at two time points: early phase (4–5 min) and late phase (10–15 min) of the session. During early phase, both *srpk3* KO and control WT fish showed social interaction behavior by staying in zone 1, close to the social cue fish. However, at late phase *srpk3* KO fish started to lose this socially interactive behavior, which might reflect the “poor attention span” exhibited by some of the patients. To investigate social interaction in zebrafish, we divided the tank into four zones (1, 2, 3, and 4). One section of the tank contained the test fish, including *srpk3* wild type or KO sibling, while the other section contained social cue fish. Three wild type fish were used as social cue. A transparent acrylic plate separated the two sections to allow for fish interaction. Our results showed that WT siblings tended to stay in zone 1 and interacted with the cue fish frequently. In contrast, *srpk3* KO zebrafish showed significantly less interaction with the cue fish and instead explored the whole tank, including zones 2, 3, and 4 (Fig. 5C–E). Based on these observations, we hypothesized that impaired social interaction may be due to some neurological defects in the brain, although we could not detect significant changes in the expression of neuronal makers at early developmental stages. Therefore, we proceeded with brain sectioning to analyze anatomical defects in the zebrafish adult brain.

### Reduced valvular cerebelli in adult srpk3 KO zebrafish

**Fig 6:**
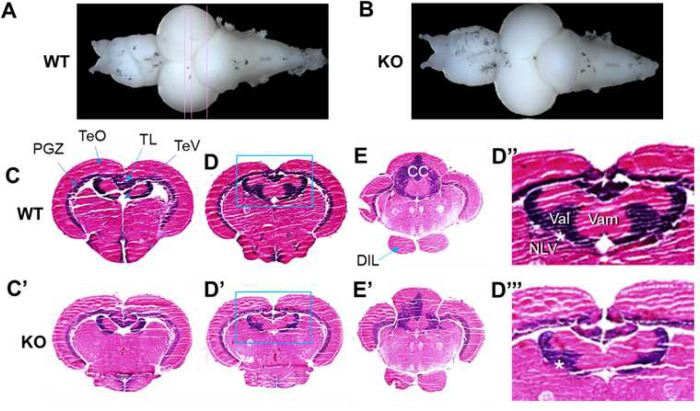
Reduced size of the valvular cerebelli in adult brain of *srpk3* KO zebrafish. **A, B** Representative whole brains dissected from a control WT and a KO zebrafish. Doral view, anterior is to the left. **C-D”’** Anatomical analysis of brain sections after H-E stain. Frontal sections at three different levels indicated in (**A**). **D”-D”’** Enlargement of valvular cerebelli region in the inlets in **D** and **D’**. Asterisk indicates a structural connection between the nucleus lateralis valvulae (NLV) and the valvular cerebelli. DIL, diffuse nucleus of the inferior lobe; PGZ, periventricular gray zone of optic tectum; TeO, tectum opticum; TeV, tectal ventricle; TL, torus longitudinalis; Val, lateral division of valvular cerebelli; Vam, medial division of valvular cerebelli; NLV, nucleus lateralis valvulae; CC, corpus cerebelli.

Although we observed impaired social interaction in adult *srpk3* KO zebrafish, we could not find significant difference in growth and body size between WT and *srpk3* KO siblings. We then tried to examine whether brain size was affected in *srpk3* KO zebrafish, given that we had previously observed reduced brain size in a zebrafish autism model [16]. We wanted to identify how *srpk3* was involved in maintaining anatomical structures of the brain. In dissected adult whole brains, we did not observe any significant difference in the overall size of dissected brains between WT and *srpk3* KO siblings (Fig. 6A,B). Next, we performed brain sections of 7 μm in wild type and KO zebrafish, performed H & E staining, and examined the brain structures in detail. To our surprise, we noticed a significant reduction in the specific brain region, so called the valvular cerebelli in fish, which is suggested to be functionally equivalent to the pontine nuclei in mammals (Fig. 6C–D”’). ImageJ analysis showed that the relative size of valvular cerebelli in the whole brain of *srpk3* KO zebrafish was reduced to 50%, 59%, and 41% from that of WT zebrafish in three different sections in Fig. 6C–E’.

## DISCUSSION

In this study, we provided multiple lines of evidence for the role of *SRPK3* in XLID. We identified novel *SRPK3* mutations segregating with the phenotype in three independent families. Using AI-based structural protein modelling, we described the effects of the missense mutations on SRPK3 structure and how they could disrupt normal SRPK3 functioning. We described for the first time early human embryonic and fetal brain expression patterns of SRPK3 and its correlation to the brain phenotypes observed in the neuroimaging of our patients. Finally, using histological analysis, social interaction assays, and modelling oculomotor behavior, we recapitulated the human phenotype in a novel *srpk3* zebrafish knockout model.

One critical cellular activity is processing of pre-mRNAs which is essential for gene expression and production of multiple isoforms from a single pre-mRNA molecule. This function is performed by the spliceosome, a complex comprised of many components [30]. SR proteins, members of a protein family containing an arginine/serine-rich (RS) domain, play a crucial role in spliceosome function [31]. However, phosphorylation of SR proteins by SR protein-specific kinases (SRPKs) is required for SR protein function [7]. In mice, *Srpk3* is mainly expressed in heart and skeletal muscles and is supposed to have a role in muscle development. Although myopathy was reported in a study of *Srpk3* KO mouse line [32], it was negatively identified in high-throughput myopathy phenotype screening of over 4,000 KO mice. *Srpk3* KO mice developed normally and showed no clinical signs of muscle weakness [33]. In our study, no myopathy phenotype was observed in *srpk3* KO zebrafish (Fig. S5). However, recently, a new function of SRPK2 was reported in synaptic vesicle and neurotransmitter release [7]. In addition, association of Srpk3 protein expression was described in a mouse model of Parkinson’s disease [34].

Since SRPKs have not been well characterized, the identification of missense mutations in *SRPK3* predicted to be pathogenic is a significant discovery. The first variant (H159D) was located in the first protein kinase domain and the second (T458N) and third (E529K) were located in the second protein kinase domain, suggesting a possible effect on kinase activity. Although, a phosphorylation assay is required to verify this prediction in further studies, molecular modeling by Alphafold showed that H159D and T458N variations caused protein misfolding and that the E529K variation could affect protein–protein interaction-mediated target protein recognition of SRPK3.

### Eye movements and intellectual disability

For visual attention and/or perception, there are two major functions of the eye – fixation and tracking. Fixation is the act of positioning the target object into the fovea. Tracking is the ability to fixate on objects while they are moving. Efficient eye movement can be related to learning problems and intellectual disability [21–23]. Convergence insufficiency is characterized by a decreased ability to converge the eyes and maintain binocular fusion while focusing on a near target. Smooth pursuit allows the eyes to slowly follow a moving target. It is common to find this function underdeveloped in children with learning disabilities. Recently, with sophisticated technologies, more advanced eye tracking methods have been developed. For example, dyslexic children showed lack of pursuit performance with an elevation in catchup saccades and decrease in gain values [35]. In schizophrenic patients, eye movement studies have revealed cognitive dysfunction as well as a link between the genetics of physiological characteristics and smooth pursuit eye movements [21,22]. Similarly, high functioning individuals with autism showed deficit in eye movements when compared to healthy individuals of the same age group [36].

### Swim bladder defects in zebrafish models of neural diseases

The swim bladder is an air-filled organ that is important for maintaining neutral buoyancy in many fish. Primary inflation of the swim bladder by surface air gulping is a critical developmental event required for free swimming and survival [37]. Zebrafish have a critical window of time during which primary inflation must occur by gulping a small gas bubble from surface air. Air gulping is accomplished by a stereotyped swimming toward the surface termed ‘swim-up behavior’ which involves multiple upward movements toward the water surface. Swim-up behavior is a very complex procedure integrating the function of multiple physiological systems, including gravity sensing, muscle development, neurological, and/or visual acuity [38]. In our previous studies, we observed swim bladder defects in several zebrafish KO models of neurodevelopmental disorders; XLID with spasticity [39], Armfield XLID syndrome [40], and ataxia [41].

### Oculomotor behavior and neural circuit function

Loss of *srpk3* resulted in a notable deficit in eye movements, or oculomotor behavior. This is most clearly seen in the frequency of spontaneous eye movements and reset time after eye movement in *srpk3* KO zebrafish relative to controls. These oculomotor behaviors observed in KO zebrafish may arise from a deficit in the circuit connectivity within the integrator for supporting and coordinating persistent firing [42,43]. Delayed reset time in KO zebrafish may partly arise from defects in the integrator pathway, which is necessary to control a smooth ramp of eye position [44].

Deviations of eye movement control are established intermediate neurophysiological phenotypes for psychotic disorders. Comparable eye movement deficits have been demonstrated in psychotic disorders indicating disturbances in brain systems subserving inhibitory control and pursuit initiation and maintenance. However, the genetic factors underlying eye movement are largely unknown. Recent genetic studies using eye movement phenotypes have predominantly focused on mutations in candidate genes for schizophrenia disease risk [45].

### Reduction in the size of valvular cerebelli in KO zebrafish

It is well known that retinal projections are topographically arranged in the optic tectum (superior colliculus in mammals) in vertebrates. Somatosensory impulses are conveyed from the optic tectum to the cerebellum. The cerebellum plays a major role in motor control and is involved in cognitive and emotional functions. The cerebellum of fish consists of the valvula cerebelli, corpus cerebelli, and caudoventral cerebellar regions. The nucleus lateralis valvulae (NLV) is a cerebellar relay nucleus in a mesorhombencephalic area, which is likely to be functionally equivalent to the pontine nuclei in mammals [46]. Thus, the retino-pretectal-NLV-valvular-cerebellar corpus pathway may play a role in visual and somatosensory information processing. In this study, we observed reduced size of valvula cerebelli, NLV and their connection in *srpk3* KO zebrafish. Although there is structural difference between the human and fish brain, there may be a conserved role of these structures. Recent studies provide molecular insights into the new role for the cerebellum in cognitive behaviors, including modulating dopaminergic reward circuits, language, and social behavior. They identified a conserved cell type set that forms an archetypal cerebellar nucleus as the unit of cerebellar nuclei organization [47]. In zebrafish, it is suggested that there is a functional difference of the valvular cerebelli; for example, non-locomotor functionality, compared to the corpus cerebelli which has a main role in locomotion [48]. Thus, future studies that selectively probe valvular cerebelli activity will provide further insights into the regional- and class-specific roles of nonlocomotory information processing of the cerebellar circuitry.

In patients, from neuroimaging data, we observed agenesis of the corpus callosum and colpocephaly. The corpus callosum (Latin for “tough body”), also known as the callosal commissure, is a wide and thick nerve tract. The corpus callosum is found only in placental mammals while being absent in other vertebrates, including fish. Agenesis of the corpus callosum is a rare congenital disorder that is one of the most common brain malformations observed in human beings.

Patients with colpocephaly have various degrees of motor disabilities, visual defects, spasticity, and moderate to severe intellectual disability. Often colpocephaly occurs as a result of hydrocephalus. Hydrocephalus is the accumulation of cerebrospinal fluid (CSF) in the ventricles of the brain. Dilated ventricles that include colpocephaly, are frequently seen in patients with agenesis of the corpus callosum. The reason for this is unclear but may be due to the loss of brain mass. The enlarged ventricles can in part be explained by reduced brain mass related to the agenesis of the corpus callosum. In other words, when some parts of the brain are not developed or underdeveloped, the space left by loss of brain matter or structure is filled by CSF, resulting in dilatation of ventricles. There is still controversy about the exact reasons for ventricular dilatation in agenesis of the corpus callosum [49]. No obstruction of CSF pathways is present and the hydrocephalus in these cases is not usually associated with raised intracranial pressure. The choroid plexus (CP) is a highly vascularized structure in the brain which helps with synthesis, secretion, and circulation of CSF. The structural and functional implications of the CP in brain diseases have been neglected for many years; however, in recent years the CP has gained attention as a neuroanatomical structure directly linked to neurodevelopmental and neuropsychiatric disorders [50]. Since *SRPK3* is highly expressed in the CP epithelium, it will be interesting to examine whether *SRPK3* is directly involved in CP development and function in future studies.
